# Phenylbutyrate and Dichloroacetate Enhance the Liquid-Stored Boar Sperm Quality via PDK1 and PDK3

**DOI:** 10.3390/ijms242317091

**Published:** 2023-12-04

**Authors:** Zhihua Guo, Yan Zhang, Anqi Huang, Qingyong Ni, Changjun Zeng

**Affiliations:** 1 State Key Laboratory of Swine and Poultry Breeding Industry, College of Animal Science and Technology, Sichuan Agricultural University, Chengdu 611134, China; jyjgzga@163.com (Z.G.); yanzhang@sicau.edu.cn (Y.Z.); niqy@sicau.edu.cn (Q.N.); 2 Key Laboratory of Livestock and Poultry Multi-Omics, Ministry of Agriculture and Rural Affairs, College of Animal Science and Technology, Sichuan Agricultural University, Chengdu 611134, China; 3 Farm Animal Genetic Resources Exploration and Innovation Key Laboratory of Sichuan Province, College of Animal Science and Technology, Sichuan Agricultural University, Chengdu 611134, China; 4 College of Animal Science and Technology, Sichuan Agricultural University, Chengdu 611134, China; 5 College of Life Science, Sichuan Agricultural University, Ya’an 625014, China; huangaqii@sicau.edu.cn

**Keywords:** boar, liquid storage sperm, PDKs, DCA, 4-PBA

## Abstract

Artificial insemination (AI) with liquid-stored semen is the most prevalent and efficient assisted reproduction technique in the modern pork industry. Pyruvate dehydrogenase complex component X (PDHX) was demonstrated to be associated with sperm metabolism and affected the boar sperm viability, motility, and fertility. Pyruvate Dehydrogenase Kinases (PDKs) are the key metabolic enzymes that regulate pyruvate dehydrogenase complex (PDHC) activity and also the conversion from glycolysis to oxidative phosphorylation. In the present study, two PDK inhibitors, Dichloroacetate (DCA) and Phenylbutyrate (4-PBA), were added to an extender and investigated to determine their regulatory roles in liquid-stored boar sperm at 17 °C. The results indicated that PDK1 and PDK3 were predominantly located at the head and flagella of the boar sperm. The addition of 2 mM DCA and 0.5 mM 4-PBA significantly enhanced the sperm motility, plasma membrane integrity (PMI), mitochondrial membrane potential (MMP), and ATP content. In addition, DCA and 4-PBA exerted their effects by inhibiting PDK1 and PDK3, respectively. In conclusion, DCA and 4-PBA were found to regulate the boar sperm metabolic activities via PDK1 and PDK3. These both can improve the quality parameters of liquid-stored boar sperm, which will help to improve and optimize liquid-stored boar semen after their addition in the extender.

## 1. Introduction

Artificial insemination (AI) is a widely used breeding technology in commercial sow breeding and is maximally performed with liquid-stored semen [1]. At present, more than 99% of AI in the boar industry is being carried out with semen stored at 15–20 °C for 1 to 5 days. In addition, 85% of all the inseminations are performed on the first or second day of semen collection [2]. However, the continuous metabolic activities of sperm under liquid storage conditions lead to the utilization of available nutrients and accumulation of their metabolic end products, which results in decreased sperm viability and hence semen quality [3,4]. Therefore, semen kept at room temperature for 1–2 days does not support transportation in remote areas for an extended period of time and results in a decreased conception rate and litter size. So, prolonging the storage time with a suitable extender might increase the economic benefit and reproductive efficiency [5].

It is a well-known fact that the motility and viability of mammalian sperm depend on available energy [6]. Sperm utilizes substances dissolved in seminal plasma and cytoplasm for the purpose of energy production. These dissolved substances play essential roles in the maintenance of sperm motility, viability, and fertility [7]. However, due to the low content of endogenous sugars, sperm rely mainly on exogenous sugars to fulfill their energy requirements during liquid storage [8]. Usually, diluents contain glucose as a major source of energy for sperm. In general, the composition of the extender affects the viability of stored boar spermatozoa [9]. Glycolysis and oxidative phosphorylation (OXPHOS) are two crucial pathways that produce energy for boar sperm [10]. Under anaerobic conditions, pyruvate produced by glycolysis is converted to lactate by lactate dehydrogenase (LDH). However, under aerobic conditions, pyruvate dehydrogenase complex (PDHC) facilitates the oxidative decarboxylation of pyruvate to generate acetyl-CoA, which is a substrate of the tricarboxylic acid (TCA) cycle [11]. PDHC is critical enzyme which connects glycolysis with oxidative phosphorylation [12]. PDHC is a multi-enzyme complex consisting of PDHA, DLAT, and DLD [13]. Pyruvate dehydrogenase (PDH) is one of the catalytic subunits of PDHC and has been correlated with ATP synthesis and mitochondrial membrane potential in mice testicular tissue [14]. Similarly, PDHC was found to be essential for human testicular tissues [11]. PDHC and its E3 subunit (dihydrolipoamide dehydrogenase (DLD)) have been reported to regulate intracellular lactate, pH, and calcium in hamster sperm [15]. Wang et al. [16] showed that pyruvate dehydrogenase complex component X (PDHX) was positively correlated with boar sperm motility and fertility.

PDHC activity is mainly catalyzed by four pyruvate dehydrogenase kinases (PDK1–4) [17]. These PDKs phosphorylate and inactivate PDHC, thus inhibiting the oxidative metabolism of pyruvate in mitochondria and affecting cellular energy production [18]. PDKs regulate oxidative stress and apoptosis along with energy metabolism. Their abnormal regulation presumably contributes to diabetes, obesity, cancer, ischemia, and metabolic acidosis [19,20,21,22,23]. Oxidative stress and mitochondrial dysfunction in the ovary are hypothesized to be due to the elevated expression of PDK1 [24]. Imanaka et al. showed that oocytes maintain higher rates of ATP production through the TCA cycle/OXPHOS system by downregulating PDK and activating PDH [25]. Dichloroacetate (DCA), as a specific inhibitor of PDK [26], promotes pyruvate metabolism by activating PDHC [27]. The ability of DCA to reduce intracellular lactate concentrations and enhance the mitochondrial oxidative metabolism by inhibiting PDKs has also been observed in numerous in vitro and in vivo studies [28]. 4-PBA is present in different human organs and tissues at lower concentrations [29], just like vitamin B1 [30]. 4-PBA has recently gained importance as it treats various disorders and also acts as a PDK inhibitor. According to different studies, 4-PBA interacts with PDKs [31,32] and reduces the severity of lactic acidosis almost similar to DCA [33]. A previous study of single-cell RNA sequencing revealed that PDK1, PDK2, and PDK3 are highly expressed in male germ cells whereas PDK4 showed very little [https://www.proteinatlas.org]. However, the effects of DCA and 4-PBA on sperm functional activities are unknown.

Our previous study indicated that the activation of PDHX could significantly improve boar sperm motility and survival [16]. However, the effects of PDKs on the regulation of PDH and hence sperm energy metabolism, motility, and survival still need to be deciphered. Therefore, this study aims to evaluate the effects of DCA and 4-PBA on the boar sperm motility, plasma membrane integrity, mitochondrial membrane potential, and ATP content during liquid storage and to investigate the molecular mechanism behind them.

## 2. Results

### 2.1. Addition of DCA or 4-PBA Improve Liquid-Stored Boar Sperm Viability

The effects of different concentrations of DCA and 4-PBA on the boar sperm viability are shown in Table 1 and Table 2. The results showed that a significant increase (*p* < 0.05) in viability was observed from 48 to 72 h in the 2 mM DCA treatment and 24 to 96 h in the 0.5 mM 4-PBA compared with their respective control groups. However, no difference (*p* > 0.05) was found between the 2 mM DCA group and the control group at 96 h, 120 h, and 144 h. In particular, the sperm viability in the 2 mM DCA treatment was higher (*p* < 0.05) than that of the other treatment groups from 48 to 96 h. Similarly, the 0.5 mM 4-PBA treatment group also showed a significantly higher (*p* < 0.05) sperm viability than the other treatment groups from 48 to 96 h. Therefore, the addition of 2 mM DCA and 0.5 mM 4-PBA was found to maintain the sperm viability better than the other concentrations.

### 2.2. Simultaneous Addition of 2 mM DCA and 0.5 mM 4-PBA Has No Synergistic Effect on Liquid-Stored Boar Sperm Viability

To determine whether the addition of DCA and 4-PBA has a synergistic effect, 2 mM DCA and 0.5 mM 4-PBA were simultaneously added. The results showed that the simultaneous addition of 2 mM DCA and 0.5 mM 4-PBA significantly increased (*p* < 0.05) the sperm viability compared with the control group at 48 h. However, no significant differences were observed among the DCA, 4-PBA, and DCA + 4-PBA treatment groups (*p* > 0.05) (Table 3).

### 2.3. Addition of 2 mM DCA and 0.5 mM 4-PBA Affect Boar Sperm Plasma Membrane Integrity (PMI), Motility, Mitochondrial Function, and ROS Level

**Figure 1:**
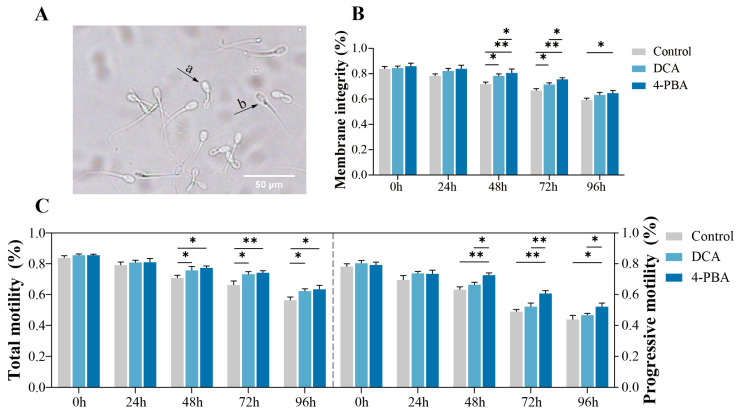
Effects of addition of 2 mM DCA and 0.5 mM 4-PBA. on boar sperm quality. (**A**) A Hypo-osmotic swelling test (HOST) assay assesses the plasma membrane integrity. a: sperm with intact plasma membrane, b: sperm with damaged plasma membranes; (**B**) plasma membrane integrity of sperm; (**C**) total motility and progressive motility of sperm. The data are representative of at least three independent experiments (mean ± SEM). “*” indicates statistical significance at *p* < 0.05 and “**” indicates statistical significance at *p* < 0.01.

The maintenance of plasma membrane integrity (PMI) is essential for sperm viability and functionality. The results indicated that, compared with the control, the PMI was significantly increased (*p* < 0.05) after the addition of 2 mM DCA and 0.5 mM 4-PBA at 48–72 h and 48–96 h, respectively (Figure 1B). Moreover, the addition of 0.5 mM 4-PBA showed a significantly higher (*p* < 0.05) PMI than the 2 mM DCA treatment from 48–72 h.

The addition of 2 mM DCA and 0.5 mM 4-PBA significantly (*p* < 0.05) improved the total and progressive motilities of the sperm from 48–96 h (Figure 1C). Furthermore, the supplementation of 0.5 mM 4-PBA showed a significantly higher (*p* < 0.05) progressive motility from 48–96 h compared to the 2 mM DCA treatment.

**Figure 2:**
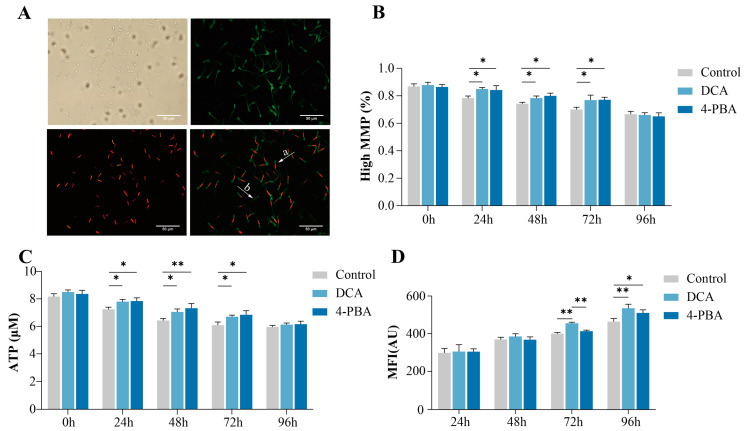
Effects of DCA or 4-PBA on boar sperm mitochondrial function and ROS levels. (**A**) Assessment of the mitochondrial membrane potential (ΔΨm) by a JC-1 staining assay. a: high ΔΨm, b: low ΔΨm; (**B**) mitochondrial membrane potential of sperm; (**C**) ATP concentration of sperm; (**D**) DCF fluorescence levels are generated by oxidation of non-fluorescent DCFH. The *Y*-axis represents the mean fluorescence intensity (MFI). The data are representative of at least three independent experiments (mean ± SEM). “*” indicates statistical significance at *p* < 0.05 and “**” indicates statistical significance at *p* < 0.01.

To investigate the effects of DCA and 4-PBA on the sperm mitochondrial membrane potential (ΔΨm), we evaluated the ΔΨm and ATP contents (Figure 2). Mitochondrial membrane potential is a good indicator of the ATP synthesizing ability of sperm. The results showed that the ΔΨm and ATP content significantly increased (*p* < 0.05) in the DCA and 4-PBA treatment groups as compared to their control groups from 24 to 72 h; however, no differences (*p* > 0.05) were observed between the DCA and 4-PBA groups (Figure 2B,C). Similarly, we did not observe any significant differences (*p* > 0.05) in the sperm mitochondrial membrane potential and ATP contents among the control, DCA, and 4-PBA groups at 96 h.

ROS are produced as a result of cellular metabolism that induce polyunsaturated fatty acids’ peroxidation and damage the sperm plasma membrane. The results indicated that there were no significant differences (*p* > 0.05) in the levels of ROS among the control and treatment groups within 48 h (Figure 2D). However, the mean fluorescence intensity (MFI) of DCF was significantly increased (*p* < 0.05) in the DCA group as compared to the control group at 72 and 96 h. Moreover, the MFI significantly increased (*p* < 0.05) at 96 h in the 4-PBA group as compared to the control group.

### 2.4. Expression of PDKs in Boar Sperm

**Figure 3:**
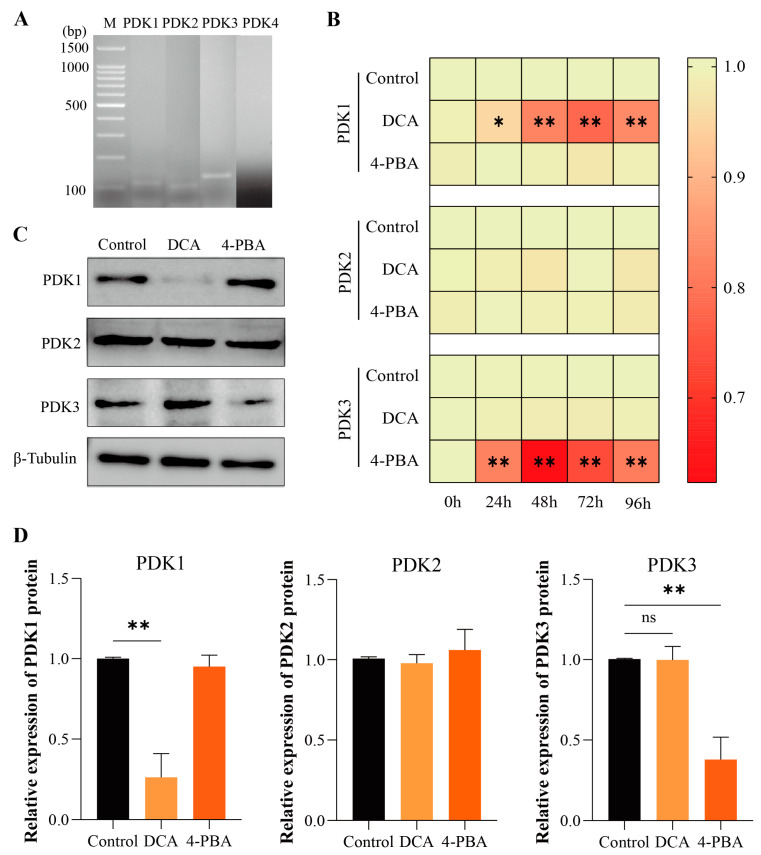
Expression of PDKs in boar sperm. (**A**) PDK1, PDK2, PDK3, and PDK4 expression in sperm; (**B**) heatmap of relative mRNA expression levels of PDK1, PDK2, and PDK3 in sperm treated with DCA or 4-PBA for 0 h to 96 h. The *X*-axis represents the processing time, and the *Y*-axis represents the relative gene expression; (**C**) the Western blot identified PDK1, PDK2, and PDK3 in sperm treated with DCA or 4-PBA for 48 h; (**D**) the relative protein levels of PDK1, PDK2, and PDK3 in sperm treated with DCA or 4-PBA at 48 h. “*” indicates statistical significance at *p* < 0.05 and “**” indicates statistical significance at *p* < 0.01, ns, no significant.

To determine whether all PDKs are expressed in boar sperm, the mRNA expression of PDKs in the sperm was further evaluated by RT-PCR. The results showed the expression of PDK1, PDK2, and PDK3 in the boar sperm. Moreover, no expression of PDK4 was observed in the boar sperm (Figure 3A).

### 2.5. Addition of DCA and 4-PBA Inhibit the Expressions of PDK1 and PDK3 in Boar Sperm

The RT-qPCR results showed that the expression of PDK1 in the DCA group was significantly decreased (*p* < 0.05 at 24 h, and *p* < 0.01 at 48–96 h) than that in the control group (Figure 3B). However, no significant differences (*p* > 0.05) were observed in the expression of PDK2 and PDK3 between the DCA and control groups. Conversely, the expression of PDK3 was significantly decreased (*p* < 0.01) in the 4-PBA group compared to the control group from 24 to 96 h. In contrast, no significant differences (*p* > 0.05) were observed in the expression of PDK1 and PDK2 between the 4-PBA and control groups. In addition, the Western blot analysis (Figure 3C) further indicated that the protein level of PDK1 was significantly decreased (*p* < 0.01) at 48 h in the DCA group compared to the control group. In contrast, no significant differences (*p* > 0.05) were observed in the expression of PDK2 and PDK3 between the DCA and control groups (Figure 3D). Compared with the control, the protein level of PDK3 in the 4-PBA group was significantly decreased (*p* < 0.01) at 48 h, whereas no significant differences (*p* > 0.05) were observed in the expressions of PDK1 and PDK2 between the 4-PBA and control groups (Figure 3D).

### 2.6. Localization of PDK1 and PDK3 Proteins in Boar Sperm

**Figure 4:**
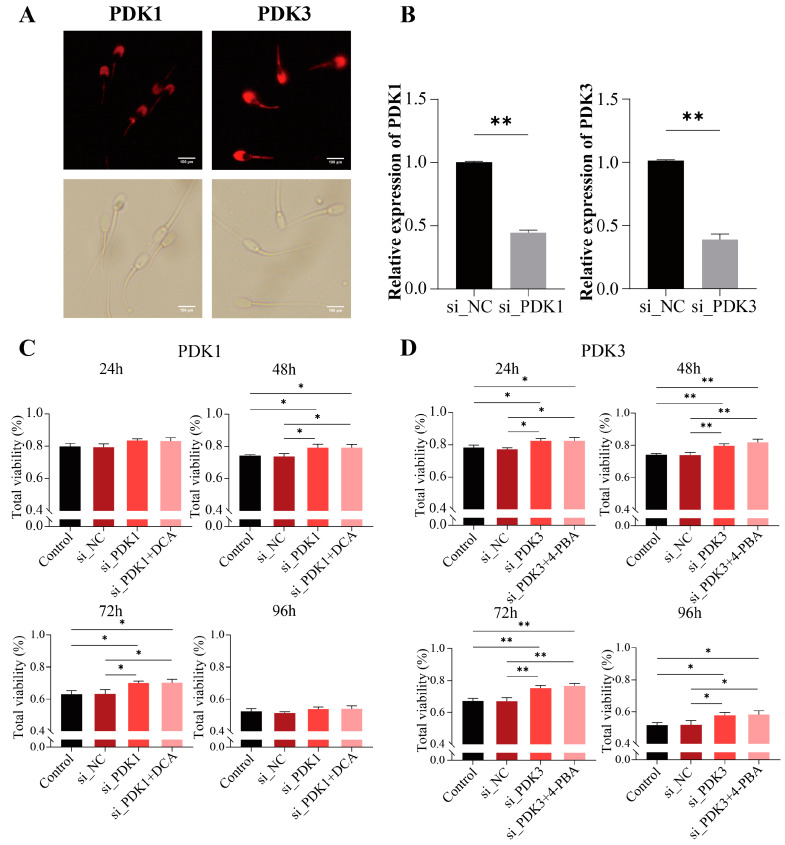
Knockdown of PDK1 and PDK3 improves boar sperm viability. (**A**) Localization of PDK1 and PDK3 in sperm; (**B**) the relative mRNA expression of PDK1 and PDK3 in si-PDK1 and si-PDK3; (**C**) sperm viability of si-PDK1 and further treated with DCA from 24 to 96 h; (**D**) sperm viability of si-PDK3 was further treated with 4-PBA from 24 to 96 h. “*” indicates statistical significance at *p* < 0.05 and “**” indicates statistical significance at *p* < 0.01.

To determine the localization of PDK1 and PDK3 in the boar sperm, we incubated the sperm with anti-PDK1 and PDK3 antibodies. The results showed the distribution of PDK3 on the head and tail of the boar sperm. However, PDK1 was found to be mainly distributed in the head region with a lesser distribution in the middle piece and tail of the boar sperm (Figure 4A).

### 2.7. Knockdown of PDK1 and PDK3 Improves Boar Sperm Viability

We knocked down PDK1 (si-PDK1) or PDK3 (si-PDK3) to verify the roles of PDK1 and PDK3 in the boar sperm and their regulation by DCA and 4-PBA. The results indicated that the knocking down of PDK1 (si-PDK1) and PDK3 (si-PDK3) significantly (*p* < 0.01) decreased the mRNA expressions of PDK1 and PDK3, respectively (Figure 4B). The total viability of the boar sperm was significantly increased (*p* < 0.05) after electro-transfection with si-PDK1 at 48–72 h (Figure 4C). Similarly, after 48 and 72 h, the sperm viabilities were significantly higher (*p* < 0.05) after the knockdown of PDK1 (si-PDK1) in the DCA-treated or untreated sperms compared with the si-NC and control groups. In contrast, no significant difference (*p* > 0.05) in the sperm viability was observed between the groups either treated or not treated with DCA (Figure 4C). Similarly, the sperm viability after the knockdown of PDK3 (si-PDK3) with or without the 4-PBA treatment was significantly higher (*p* < 0.01) at 24 to 72 h and significant (*p* < 0.01) at 96 h (Figure 4D). However, no significant difference (*p* > 0.05) in the sperm viability was observed between the si-PDK3 groups with or without 4-PBA (Figure 4D). Therefore, these results indicated that DCA and 4-PBA exert their effects on the sperm viability by inhibiting PDK1 and PDK3, respectively.

## 3. Discussion

A previous study showed that DCA and 4-PBA affect the glucose metabolism in cancer cells by regulating the mRNA and protein expressions of PDKs [34]. However, no study has reported the effects of the addition of DCA and 4-PBA on boar sperm and their interaction with PDKs. This study provides novel evidence that DCA and 4-PBA are directly linked with PDKs in boar sperm and regulate their energy metabolism and viability during liquid storage.

DCA is a structural analog of pyruvate dehydrogenase complex (PDHC) that can bind to the pyruvate-binding site in the center of the R domain to inhibit the activity of PDKs [34]. By blocking PDKs, DCA can activate PDHC and hence increase the activity of the mitochondrial respiratory chain and ATP synthesis [35]. Similarly, Chen et al. treated 24 mice with 4-PBA for a fortnight and observed an improvement in mitochondrial function [36]. It is well known that sperm characteristics, such as motility, are important to ensure fertility potential and play a crucial role in the prediction of fertility. The combined aspects of sperm function are prerequisites for normal in vivo fertilization and early embryonic development [37,38]. In the present study, the boar sperm quality and mitochondrial function were significantly improved after the treatment with DCA and 4-PBA, indicating that DCA and 4-PBA had a promoting effect on fertility. However, further studies are needed to explain the role of DCA and 4-PBA in farrowing the rate and litter size to elucidate the long-term effects of DCA and 4-PBA on sperm preservation. However, the effects of both additives on the sperm parameters became less pronounced when the semen was stored for 96 h. The elevated levels of ROS provide a plausible explanation during the longer storage. ROS are by-products of oxygen metabolism inside sperm mitochondria [39]. The accumulating evidence indicates that the threshold level of ROS is a requisite for normal sperm function [40]. At physiological concentrations, ROS induce sperm capacitation and acrosomal reaction, which enable sperms to penetrate zona pellucida during fertilization [41]. Conversely, excessive ROS production causes oxidative damage to biomolecules and poses detrimental effects on the overall sperm quality (DNA integrity, membrane stability, energy production, motility, capacitation) [42,43,44]. In this study, the increase in the ATP levels was followed by an abnormal increase in ROS after the DCA or 4-PBA treatment similar to studies conducted on cancer cells [45,46,47]. Interestingly, Zhao et al. [48] found that DCA can relieve oxidative stress through the PDK2-PDH-Nrf2 channel. Similarly, Yang et al. [49] found that 4-PBA significantly inhibited oxidative stress, reduced the shock-induced oxidative stress index, such as the production of reactive oxygen species, and increased the levels of antioxidant enzymes such as superoxide dismutase, catalase, and glutathione.

External factors like the seminal plasma composition and availability of oxygen regulate the homeostasis of metabolic pathways in sperm, which suggests that a mere change in the external environment may disturb the metabolism of sperm [50]. Glycolysis and oxidative phosphorylation are two of the main pathways that control the sperm function and its fertilizing ability [51]. However, little is known about the exact metabolic pathway that is used by boar sperm for the purpose of ATP production and hence required for its movement and viability. The PDHC/PDK axis is known to be an essential part for the regulation of glucose metabolism [52]. PDKs are known to affect cellular energy production during pyruvate metabolism via the regulation of PDHC phosphorylation [12]. PDKs comprise four members (PDK1–PDK4) in the mitochondrial matrix [53]. Although all PDKs exhibit about a 70% structural similarity, they still have different relative affinities for binding to PDHC (PDK > PDK1 ≈ PDK2 > PDK4) [54]. Moreover, these four PDKs also exist in mammalian tissues with different tissue-specific distributions and kinetic parameters [55]. PDK2 is most widely distributed in the heart, liver, and kidney, while PDK4 is abundantly expressed in pancreatic islets and skeletal muscles. PDK3 and PDK1 show limited tissue distribution and PDK3 is more expressed in testes and lungs [56]. In this study, the RT-PCR results showed that all PDKs (PDK1, PDK2, and PDK3) except PDK4 are expressed in sperm. Thus, we hypothesized that PDK1–3 might be the PDKs that play a crucial role in boar sperm viability regulation.

Studies have demonstrated that DCA [57] and 4-PBA [33] can inhibit PDKs in tumor cells and can restore the mitochondrial metabolism. 4-PBA is known to affect only PDK1, PDK2, and PDK3. However, unlike 4-PBA, DCA can bind to all PDKs [30]. Our results show that DCA treatment inhibits PDK1 expression in boar sperm, which is similar to the results reported by Hur et al. [58] in gastric cancer cells. Similarly, DCA was reported to inhibit PDK3 in melanoma cells [59]. In the present study, 4-PBA was found to inhibit PDK3 in boar sperm, whereas another study on fibroblasts reported the inhibition of PDK2 by 4-PBA [31]. This difference is not surprising because the targets of DCA and 4-PBA could vary across different cell types [60,61]. Meanwhile, we observed that the inhibition of PDK1 (si-PDK1) or PDK3 (si-PDK3) followed by the addition of DCA or 4-PBA did not increase the sperm viability. Therefore, it is confirmed that DCA inhibits PDK1 and 4-PBA inhibits PDK3. Moreover, immunofluorescence localization showed that PDK1 and PDK3 were distributed in the head and midpiece of the boar sperm. It is known that mitochondria are mainly distributed in the midpiece of sperm, which helps to elucidate the role of PDK1 and PDK3 in the boar sperm metabolism.

PDK inhibitors usually regulate PDK activity by acting on four binding sites: a pyruvate binding site, nucleotide binding site, lipoamide binding site, and allosteric site [34]. DCA is a structural analog of PDHC substrate pyruvate, which can bind to the pyruvate binding site in the center of the R domain to regulate the activity of PDKs [34]. 4-PBA binds to the allosteric site of PDKs [34]. Central to mitochondrial function is the interaction between PDKs and PDHC [62].

In conclusion, our findings provide reasonable molecular evidence and reveal a potential link between DCA/PDK1, 4-PBA/PDK3, and sperm quality. The addition of 2 mM DCA and 0.5 mM 4-PBA can improve liquid-stored boar sperm quality. Similarly, DCA and 4-PBA could be potential regulatory factors that can regulate the mitochondrial function of sperm.

## 4. Materials and Methods

### 4.1. Animal Ethics Statement

All animal procedures involving animal treatments were performed in accordance with the Institutional Animal Care and Use Committee in the College of Animal Science and Technology, Sichuan Agricultural University, Sichuan, China (under permit no. 2021202037), which conforms to the Regulations of the Administration of Affairs Concerning Experimental Animals (Ministry of Science and Technology, China, 2017).

### 4.2. Semen Collection and Treatment

**Figure 5:**
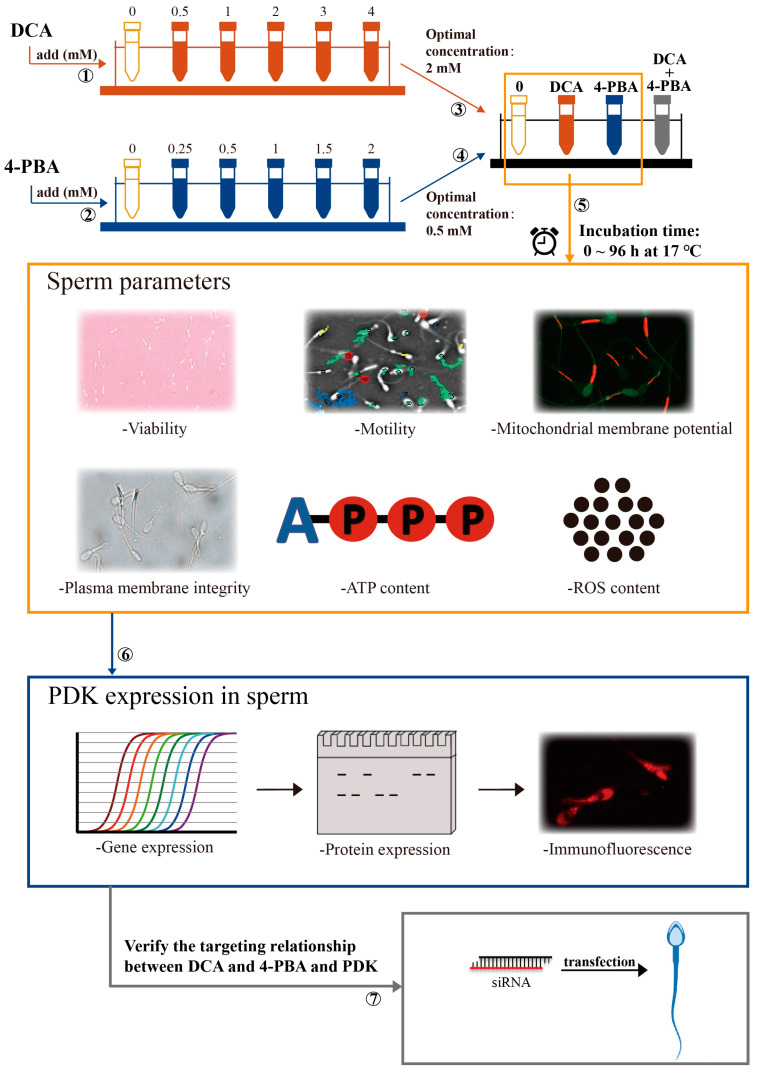
Schematic representation of the study experimental design.

The semen used in the experiments was collected from ten healthy and sexually active Large White boars. We collected the fresh ejaculates transported to the laboratory in a temperature-controlled container (17 °C). Semen samples with more than 80% motility and 85% normal morphology were used in this study, as presented in Figure 5.

All semen samples (n = 10) from each boar were pooled together, centrifuged (1500 rpm/min, 5 min), and immediately suspended in an extender (NUTRIXcell Ultra; IMV, Legrand, Paris, France) to obtain a final concentration of 1.5 × 10^9^ sperm mL^−1^. Then, we divided the semen samples into four aliquots. One aliquot was further divided into six aliquots. Different doses of DCA (Sigma-Aldrich, St. Louis, MO, USA) were then added to each aliquot to obtain the final 0, 0.5, 1, 2, 3, and 4 mM concentrations of DCA. A second aliquot was similarly further divided to obtain the final 0, 0.25, 0.5, 1, 1.5, and 2 mM concentrations of 4-PBA (Sigma-Aldrich, St. Louis, MO, USA). After the addition of different concentrations of DCA and 4-PBA, the sperm quality parameters were evaluated. The third aliquot was further divided into four aliquots and then processed as follows: (1) control: DCA and 4-PBA non-supplemented; (2) DCA: supplemented with 2 mM of DCA; (3) 4-PBA: supplemented with 0.5 mM of 4-PBA; and (4) supplemented with 2 mM DCA and 0.5 mM 4-PBA. The fourth aliquot was further divided into two aliquots to perform the knockdown of PDK1 and PDK3, from which one aliquot was transfected with 20 nM siRNA of PDK1, siRNA of PDK3, and siRNA of NC for 12 h and the other one was additionally incubated with DCA or 4-PBA after transfection. Finally, all the above semen samples were incubated for 24, 48, 72, and 96 h under the same conditions.

### 4.3. Analysis of Sperm Quality Parameters

#### 4.3.1. Sperm Viability Detection

We evaluated the sperm viability via eosin–nigrosine staining [63]. For this purpose, 20 μL of semen sample was mixed with 5 μL of eosin (Solarbio, Beijing, China) in a test tube and left for 30 s. Then, the mixture was added to with 15 μL of nigrosine stain (Solarbio, Beijing, China) for another 30 s. After incubation for 5 min at 37 °C, a smear was prepared on a glass slide and at least 200 sperm were examined by a light microscope (Yongxin Optical Co., Ltd., Zhejiang, China). Live sperm remained unstained, while dead sperms were stained pink or red.

#### 4.3.2. Sperm Motility Detection

Samples were evaluated by a computer-assisted semen analyzer (CASA, Minitube, Tiefenbach, Germany). Before measuring sperm motility, the sperm counting chamber (Leja^®^, Nieuw-Vennep, the Netherlands) was pre-heated at 37 °C on the hot stage of the system, and the CASA was debugged until the camera image was clear [64]. Five microliters of semen was dropped on the pre-heated glass slide, and five visual fields containing more than 200 sperm were selected to measure the sperm total and progressive motilities.

#### 4.3.3. Detection of Sperm Plasma Membrane Integrity

Hypo-osmotic swelling test (HOST) was used to measure the sperm plasma membrane integrity [65]. Briefly, 200 μL of the semen was mixed with 1 mL of HOST solution and incubated at 37 °C for 30 min. A light microscope (Yongxin Optical Co., Ltd., Zhejiang, China) was used to evaluate the plasma membrane status of the sperm with no less than 200 sperm per replicate. Sperm with integrated and intact plasma membranes had swollen and bent tails, while sperms with dysfunctional plasma membranes had a straight and non-swollen tail (Figure 1A).

#### 4.3.4. Detection of Mitochondrial Membrane Potential (ΔΨm)

ΔΨm was evaluated using JC-1 Mitochondrial Membrane Potential Detection Kit (Solarbio, Beijing, China), according to the manufacturer’s instructions. Briefly, the sperm were stained with JC-1 (5,5,6,6-tetrachloro-1,1,3,3-tetraethylbenzimidazolyl carbocyanine iodide) and incubated at 37 °C in the dark for 20 min. Then, the samples were centrifuged at 600× *g* at 4 °C for 5 min, and the supernatant was discarded. After that, the precipitate was resuspended in PBS, and 5 µL from this suspension was dropped on a plane glass slide and covered with cover slip. Approximately 200 sperm were observed under an epifluorescence microscope (Olympus, Tokyo, Japan). Sperm showing red fluorescence in the midpiece were considered to have a high ΔΨm (Figure 2A).

#### 4.3.5. Detection of ATP Concentration

The ATP content of sperm was assessed using the ATP Assay Kit (Nanjing Jiancheng Bioengineering Institute, Nanjing, China), according to the manufacturer’s instructions and the protocol formulated by Feng et al. [66]. Briefly, 20 µL semen and an equal volume of working solution were uniformly mixed and loaded onto a 96-well plate. Following 10 min incubation at room temperature, the luminescence signals were measured in triplicate in 96-well plates using a multimode microplate reader (Thermo Fisher Scientific, Waltham, MA, USA). The ATP content in the semen samples was calculated from an ATP standard curve.

#### 4.3.6. Evaluation of ROS Content

The amount of ROS in sperm cells was detected by DCFH-DA staining [67]. The experiment was performed by following the instructions provided in the manual of the Reactive Oxygen Species Assay Kit (S0033M, Beyotime Institute of Biotechnology, Nantong, China). Briefly, the sperm samples were centrifuged at 800× *g* for 5 min, and the supernatant was discarded. Then, the sperms were re-suspended in 200 µL working solution of Dichlorodihydrofluorescein diacetate (DCFH-DA) and incubated at 37 °C in the dark for 30 min. Sperms were rinsed three times with PBS and then re-suspended in the PBS for evaluation. A flow cytometer (FACSVerse, BD Biosciences, Franklin Lakes, NJ, USA) was used to evaluate the DCF fluorescence level generated from the non-fluorescent DCFH oxidation. We performed all the above-mentioned experiments three times to obtain three biological replicates and the results are presented as an average of these replicates.

### 4.4. Total RNA Extraction and Quantitative Real-Time PCR (RT-qPCR) Analysis

We extracted the total RNA of the sperm with Trizol LS Reagent kit (Invitrogen, Carlsbad, CA, USA), as described previously [68]. RNAs with OD260/280 values of 1.8–2.0 were selected for the subsequent reverse transcription. Evo M-MLV RT mix kit (Code no. AG11728, Hunan, China) and SYBR Green Pro Taq HS 266 qPCR kit (Code no. 11701, Hunan, China) were used for reverse transcription, according to the manufacturer’s instructions. Amplifications were performed in a CFX 96 Real-Time PCR Detection System (Bio-Rad, Hercules, CA, USA) using the master mix volume (10 µL) comprising 5 µL SYBR Green I Premix, 0.5 µL each of the forward and reverse primers, 1 µL of cDNA, and 3 µL of RNase-free water. Three biological replicates were set for each group. All primers were designed according to their counterparts in GenBank using the NCBI Primer-Blast search tool or from the published literature (Table 4). The relative mRNA quantifications were performed by comparing the genes of interest with the Glyceraldehyde-3-phosphate dehydrogenase (GAPDH), and the results were calculated using the 2^−ΔΔCT^ method [69].

### 4.5. Reverse Transcription–Polymerase Chain Reaction (RT-PCR) Analysis

The total RNA of sperm was extracted using a Trizol LS Reagent kit (Invitrogen, Carlsbad, CA, United States), as described previously [68]. RNA with the optimal OD260/280 of 1.8–2.0 was selected for the subsequent reverse transcription. Evo M-MLV RT mix kit (Code no. AG11728, Hunan, China) and 2×TransYaq-T PCR SuperMix (TransGen Biotech, AS122, Beijing, China) were used for reverse transcription, according to the manufacturer’s instructions. Subsequently, PCR products were separated using 3% agarose gel electrophoresis. The voltage was set to 150 V and the gels were run for 1 h. After electrophoresis, the glue blocks were removed, observed, and photographed with a gel imager. Three replicates were run for each group.

### 4.6. Western Blot

Western blotting analysis was carried out according to the method used by Wang et al. [70] with some required modifications. Total protein was extracted from sperm using an RIPA lysis buffer (Beyotime Biotechnology, B0013B, Shanghai, China). Total protein concentration was measured using a BCA protein detection kit (ComWin Biotechnology Co., Ltd. AR0146, Taizhou, China). The SDS-PAGE technique was employed to separate the dissolved proteins on 10% pre-casted Hepes gel (Beyotime Biotechnology, P0508S, Shanghai, China) and transferred to a PVDF membrane (Beyotime Biotechnology, FFP32, Shanghai, China). The membranes were then blocked with a blocking buffer (Beyotime Biotechnology, P0252, Shanghai, China) at room temperature for 1 h. Afterward, the membranes were incubated overnight with diluted primary antibody solutions at 4 °C. The primary antibodies used in this study were anti-PDK1 (1:1500, ABclonal, A8930, Wuhan, China), anti-PDK2 (1:1500, ABclonal, A4737, Wuhan, China), anti-PDK3 (1:1500, ABclonal, A8028, China), and β-tubulin (1:1000; Proteintec, 10094-1-AP, Wuhan, China). Notably, each incubation was an individual experiment. The membranes were subsequently washed three times with Tris-buffered saline containing 0.1% Tween-20 (TBST) and incubated with secondary antibody, viz., goat anti-rabbit IgG conjugated with HRP (1:4000, Proteintech, SA00001-2, Wuhan, China) for 1 h on a shaker at 37 °C. According to the manufacturer’s instructions, enhanced chemiluminescence (ECL) detection was then performed using the BeyoEcl Moon kit (Beyotime Biotechnology, P0018FS, Shanghai, China). Protein bands on PVDF membranes were detected using an eBlot Touch Imager (Yibote Optoelectronic Technology, Shanghai, China), and the intensities of the bands were quantified with ImageJ software. At least three independent experiments were performed.

### 4.7. Immunolocalization of PDK1 and PDK3 in Boar Sperm

Indirect immunofluorescence [70] was used to determine the localization of PDK1 and PDK3 proteins on boar sperm. For this purpose, we first fixed the sperm precipitates with 4% paraformaldehyde for 10 min, washed with PBS, and then permeabilized with 0.5% Triton X-100 (Beyotime Biotechnology, Shanghai, China). Subsequently, the samples were incubated with 5% bovine serum albumin (BSA) (Sigma-Aldrich, USA) for 30 min. After that, the samples were incubated with primary antibodies, including anti-PDK1 (ABclonal, A8930, Wuhan, China) and anti-PDK3 (ABclonal, A8028, Wuhan, China) antibodies diluted (1:100) in Western primary antibody diluent (Beyotime Biotechnology, Shanghai, China) at 37 °C for 1 h. After that, the samples were washed three times and then incubated for 1 h at 37 °C with secondary antibody (anti-rabbit IgG (H + L)). This secondary antibody was conjugated with CoraLite 594 (Proteintech, SA00013-4, Chicago, USA) and was diluted with Western secondary antibody diluent (Beyotime Biotechnology, Shanghai, China) at a ratio of 1:50. After three washings with PBST (PBS, 1% Tween, and 0.02 g glycine), sperms were observed and evaluated using a fluorescence microscope equipped with a DP70 camera (Olympus, Tokyo, Japan).

### 4.8. Sperm Electro-Transfection

Electro-transfection was performed according to Yuan’s electro-transfection method [71]. Cell Manipulation ECM-2001 (BTX, Holliston, MA, USA) was used to transfect the sperm with siRNA-targeting PDK1 and PDK3 mRNAs in order to reduce their expressions. Pulse conditions were adjusted as 4 × 300 V for 100 µs. After siRNA transfection (knockdown), sperm were stored under the same conditions used for other sperm groups, and transfection efficiency was detected at 12 h.

### 4.9. Statistical Analysis

SPSS 26.0 software was used for one-way analysis of variance (ANOVA), and all the values were expressed as mean ± standard deviation (SD). Tukey’s method was used for multiple comparisons. The relative PDK gene expression levels were quantified by the 2^−∆∆CT^ method. The gray values of protein bands were calculated using ImageJ (v.1.48). *p* < 0.05 and *p* < 0.01 were considered statistically significant.

## 5. Conclusions

This is the first study that investigates the effects of DCA and 4-PBA on boar sperm quality during liquid storage. We demonstrated that the addition of DCA and 4-PBA improved the boar sperm quality by inhibiting PDK1 and PDK3, respectively. Both PDK1 and PDK3 were found to regulate the mitochondrial function, plasma membrane stability, and ATP level. In conclusion, we propose that DCA and 4-PBA can be added in boar semen extenders to prolong the time of liquid semen storage as it results in the maintenance of the sperm viability and motility for a longer duration.

## Figures and Tables

**Table 1:** Effects of different concentrations of DCA on boar sperm viability during liquid storage.

Storage Time	DCA Treatments					
	0 mM	0.5 mM	1 mM	2 mM	3 mM	4 mM
0 h	0.92 ± 0.02 ^a^	0.90 ± 0.01 ^a^	0.91 ± 0.01 ^a^	0.91 ± 0.01 ^a^	0.89 ± 0.01 ^a^	0.86 ± 0.03 ^a^
24 h	0.82 ± 0.01 ^ab^	0.83 ± 0.01 ^ab^	0.84 ± 0.00 ^ab^	0.86 ± 0.01 ^a^	0.78 ± 0.02 ^bc^	0.76 ± 0.02 ^c^
48 h	0.74 ± 0.01 ^bc^	0.76 ± 0.01 ^b^	0.76 ± 0.01 ^b^	0.82 ± 0.01 ^a^	0.75 ± 0.02 ^bc^	0.70 ± 0.01 ^c^
72 h	0.69 ± 0.01 ^b^	0.69 ± 0.00 ^b^	0.70 ± 0.01 ^b^	0.76 ± 0.01 ^a^	0.64 ± 0.01 ^c^	0.58 ± 0.01 ^d^
96 h	0.60 ± 0.01 ^ab^	0.58 ± 0.00 ^bc^	0.58 ± 0.00 ^bc^	0.63 ± 0.02 ^a^	0.55 ± 0.01 ^cd^	0.50 ± 0.01 ^d^
120 h	0.50 ± 0.01 ^ab^	0.48 ± 0.01 ^ab^	0.49 ± 0.01 ^ab^	0.53 ± 0.00 ^a^	0.46 ± 0.02 ^b^	0.41 ± 0.01 ^c^
144 h	0.43 ± 0.01 ^a^	0.42 ± 0.01 ^a^	0.43 ± 0.01 ^a^	0.46 ± 0.01 ^a^	0.41 ± 0.01 ^ab^	0.37 ± 0.01 ^b^

Note: Different letters within the same row show significant differences (*p* < 0.05).

**Table 2:** Effects of different concentrations of 4-PBA on boar sperm viability during liquid storage.

Storage Time	4-PBA Treatments					
	0 mM	0.25 mM	0.5 mM	1 mM	1.5 mM	2 mM
0 h	0.91 ± 0.01 ^a^	0.92 ± 0.01 ^a^	0.92 ± 0.01 ^a^	0.92 ± 0.01 ^a^	0.90 ± 0.02 ^a^	0.89 ± 0.02 ^a^
24 h	0.82 ± 0.01 ^bc^	0.84 ± 0.02 ^ab^	0.87 ± 0.01 ^a^	0.82 ± 0.01 ^b^	0.82 ± 0.01 ^ab^	0.77 ± 0.01 ^c^
48 h	0.75 ± 0.01 ^bc^	0.77 ± 0.00 ^b^	0.82 ± 0.01 ^a^	0.78 ± 0.01 ^b^	0.75 ± 0.01 ^bc^	0.73 ± 0.01 ^c^
72 h	0.71 ± 0.01 ^bc^	0.72 ± 0.01 ^b^	0.79 ± 0.01 ^a^	0.73 ± 0.01 ^b^	0.67 ± 0.01 ^cd^	0.62 ± 0.01 ^d^
96 h	0.59 ± 0.01 ^bc^	0.61 ± 0.01 ^bc^	0.68 ± 0.01 ^a^	0.62 ± 0.02 ^b^	0.56 ± 0.01 ^cd^	0.53 ± 0.01 ^d^
120 h	0.51 ± 0.01 ^ab^	0.51 ± 0.03 ^ab^	0.55 ± 0.01 ^a^	0.52 ± 0.01 ^a^	0.49 ± 0.01 ^ab^	0.44 ± 0.01 ^b^
144 h	0.44 ± 0.01 ^a^	0.42 ± 0.01 ^ab^	0.46 ± 0.01 ^a^	0.44 ± 0.01 ^ab^	0.43 ± 0.01 ^ab^	0.39 ± 0.01 ^b^

Note: Different letters within the same row show significant differences (*p* < 0.05).

**Table 3:** Effect of DCA and 4-PBA treatment alone or in combination on the viability of boar sperm.

Storage Time	Treatments			
	Control	2 mM DCA	0.5 mM 4-PBA	2 mM DCA + 0.5 mM 4-PBA
0 h	0.91 ± 0.01 ^a^	0.92 ± 0.01 ^a^	0.92 ± 0.01 ^a^	0.92 ± 0.01 ^a^
24 h	0.82 ± 0.01 ^bc^	0.84 ± 0.02 ^ab^	0.87 ± 0.01 ^a^	0.82 ± 0.01 ^b^
48 h	0.76 ± 0.02 ^b^	0.82 ± 0.01 ^a^	0.83 ± 0.00 ^a^	0.81 ± 0.01 ^a^
72 h	0.71 ± 0.01 ^c^	0.75 ± 0.01 ^b^	0.79 ± 0.01 ^a^	0.74 ± 0.01 ^bc^
96 h	0.59 ± 0.01 ^bc^	0.64 ± 0.01 ^b^	0.68 ± 0.01 ^a^	0.57 ± 0.02 ^c^
120 h	0.51 ± 0.01 ^a^	0.54 ± 0.01 ^a^	0.55 ± 0.01 ^a^	0.50 ± 0.02 ^a^
144 h	0.45 ± 0.02 ^a^	0.45 ± 0.00 ^a^	0.47 ± 0.01 ^a^	0.44 ± 0.01 ^a^

Note: Different letters within the same row show significant differences (*p* < 0.05).

**Table 4:** Primer information for RT-qPCR and RT-PCR.

Gene	Sequence (5′-3′)	Tm (°C)	Size (bp)
GAPDH	F: ACCCAGAAGACTGTGGATGG	60.0	346
	R: CATGGCCTCCAAGGAGTAAG		
PDK1	F: TGAGAGCAACGATGGAGCAC	55.0	106
	R: CCTCGGTCACTCATCTTCACA		
PDK2	F: GTCTATGTCCCCTCCCACCT	60.0	100
	R: ATGGGAGAGTGAGGCTGGAT		
PDK3	F: AAGAACCGTGTCATGGGAGAG	57.0	157
	R: CTCTGAACCAATCCCACCGA		
PDK4	F: GCTGGTGACTGGTGTATCCC	55.0	138
	R: CACGCACACATTCAGGAAGC		

## Data Availability

Data are contained within the article.
